# Immunotherapy for Parkinson’s Disease and Alzheimer’s Disease: A Promising Disease-Modifying Therapy

**DOI:** 10.3390/cells13181527

**Published:** 2024-09-12

**Authors:** Anns Mahboob, Hasan Ali, AlJazi AlNaimi, Mahmoud Yousef, Mlaak Rob, Nawaf Ahmad Al-Muhannadi, Degiri Kalana Lasanga Senevirathne, Ali Chaari

**Affiliations:** Weill Cornell Medicine–Qatar, Qatar Foundation, Education City, Doha P.O. Box 24144, Qatar; anm4019@qatar-med.cornell.edu (A.M.); hoa4002@qatar-med.cornell.edu (H.A.); aaa4025@qatar-med.cornell.edu (A.A.); mmy4002@qatar-med.cornell.edu (M.Y.); mtr4001@qatar-med.cornell.edu (M.R.); naa4004@qatar-med.cornell.edu (N.A.A.-M.); dls4003@qatar-med.cornell.edu (D.K.L.S.)

**Keywords:** Alzheimer’s disease (AD), Parkinson’s disease (PD), neurodegenerative diseases (NDs), disease-modifying therapy, monoclonal antibodies (mAbs)

## Abstract

Alzheimer’s disease (AD) and Parkinson’s disease (PD) are two neurodegenerative diseases posing a significant disease burden due to their increasing prevalence and socio-economic cost. Traditional therapeutic approaches for these diseases exist but provide limited symptomatic relief without addressing the underlying pathologies. This review examines the potential of immunotherapy, specifically monoclonal antibodies (mAbs), as disease-modifying treatments for AD and PD. We analyze the pathological mechanisms of AD and PD, focusing on the roles of amyloid-beta (Aβ), tau (τ), and alpha-synuclein (α-syn) proteins. We discuss the latest advancements in mAb therapies targeting these proteins, evaluating their efficacy in clinical trials and preclinical studies. We also explore the challenges faced in translating these therapies from bench to bedside, including issues related to safety, specificity, and clinical trial design. Additionally, we highlight future directions for research, emphasizing the need for combination therapies, improved biomarkers, and personalized treatment strategies. This review aims to provide insights into the current state and future potential of antibody-based immunotherapy in modifying the course of AD and PD, ultimately improving patient outcomes and quality of life.

## 1. Introduction

Alzheimer’s disease (AD) and Parkinson’s disease (PD) represent two of the most prevalent and devastating neurodegenerative diseases (NDs) affecting the elderly population globally. The incidence of these NDs is rising at an alarming rate, placing an increasing burden on healthcare systems and economies worldwide. In 2023, an estimated 55 million people worldwide were living with dementia, with AD accounting for 60–70% of these cases. By 2050, this number is projected to triple, reaching 152 million individuals worldwide [1]. Similarly, PD affects approximately 10 million people globally, with an expected doubling of cases by 2040 [2].

The pathological hallmarks of AD and PD differ significantly, but their shared disease of burden makes them both an important area of study and our focus. AD is primarily characterized by the accumulation of amyloid-beta (Aβ) plaques and neurofibrillary tangles composed of hyperphosphorylated tau (τ) protein. These aggregates lead to widespread neuronal loss, synaptic dysfunction, cognitive decline, and memory impairment [3]. Conversely, PD is marked by the degeneration of dopaminergic neurons in the substantia nigra and the presence of Lewy bodies, which are intracellular inclusions primarily composed of alpha-synuclein (α-syn) protein. This neuronal loss results in the hallmark motor symptoms of PD, including bradykinesia, rigidity, and tremors, as well as non-motor symptoms, such as cognitive impairment and autonomic dysfunction [4].

Despite advances in our understanding of the molecular underpinnings of these diseases, current therapeutic options remain largely symptomatic, offering limited efficacy in halting or reversing disease progression. Traditional treatments for AD, such as cholinesterase inhibitors and N-methyl-D-aspartic acid (NMDA) receptor antagonists, provide modest symptomatic relief but do not address the underlying pathology [5]. Similarly, dopaminergic therapies for PD, including levodopa and dopamine agonists, improve motor symptoms but fail to modify the disease course or prevent neurodegeneration [6].

The development of antibody-based therapies has emerged as a promising approach for the disease-modifying treatment of NDs. Monoclonal antibodies (mAbs) offer the potential for the specific targeting of pathogenic proteins involved in AD and PD. In AD, mAbs targeting Aβ and τ proteins aim to reduce the formation and accumulation of toxic aggregates, thereby mitigating neurodegeneration [7]. For PD, mAbs targeting a-syn can prevent the spread of pathological aggregates and preserve neuronal integrity [8].

Our literature review aims to provide a comprehensive overview of current therapeutic strategies for AD and PD, focusing on the potential and promise of antibody-based therapies. By examining the latest research and clinical trials, we highlight the advancements, challenges, and future directions of antibody therapy for these debilitating NDs.

## 2. The Importance of ND Treatment

### 2.1. Increasing Incidence and Socio-Economic Burden

The rising incidence of AD and PD, driven by aging populations, has profound and far-reaching socio-economic implications. These debilitating NDs place a substantial burden not only on healthcare systems but also on caregivers and society.

AD is one of the most prevalent NDs, and its socio-economic impact is staggering. In recent times, experts have estimated the cost of Alzheimer’s care in the United States at USD 305 billion [9]. This figure includes direct medical expenses, such as hospital care, medication, and professional caregiving, and indirect costs like lost income and reduced productivity of patients, and it is anticipated that these costs will exceed USD 1 trillion by 2050, driven by the aging population [9]. AD not only affects the patients but also their families, who often bear the brunt of caregiving responsibilities. This caregiving role can lead to significant physical, emotional, and financial stress, further amplifying the disease’s societal impact [9]. Similarly, PD is imposing a substantial economic burden. The total cost of PD in the United States is estimated at USD 52 billion annually, including USD 25.4 billion in direct medical costs and USD 26.5 billion in indirect costs [10]. Patients with PD often require long-term care and assistance with daily activities, which can strain both public healthcare systems and private resources.

The socio-economic impact of AD and PD extends beyond direct medical costs. It encompasses lost productivity, as patients and their caregivers often must reduce work hours or leave their jobs entirely [9]. Informal care by family members who provide unpaid assistance represents a significant portion of the economic burden [11]. This informal caregiving may lead to emotional and psychological stress, resulting in health complications, high levels of stress, and depression for the caregivers themselves [11].

The increasing prevalence of NDs necessitates urgent action to develop effective treatments and interventions. Current treatments mainly focus on managing symptoms rather than curing the diseases, highlighting a significant gap in medical research and therapeutic development. Investment in research is critical to understanding the underlying mechanisms of these diseases, which could lead to breakthroughs in treatment and prevention.

### 2.2. Growing Number of Clinical Trials

Despite the rising incidence of NDs and the significant resources invested in research, there is still no cure for these debilitating conditions. Clinical trials targeting NDs have increased substantially over the past decade, with over 2000 active clinical trials focused on AD and 1400 on PD in 2023 [12]. These trials encompass a wide range of therapeutic strategies aimed at addressing the underlying pathophysiology of these diseases.

The increasing number of clinical trials signifies a concerted effort to find effective treatments for neurodegenerative diseases. This trend may result in several important outcomes: the accelerated discovery of treatments, increased knowledge and understanding of disease mechanisms, and improved clinical practices. Conducting more trials increases the probability of discovering effective therapies, potentially leading to the identification of new drugs or treatment modalities that can significantly improve patient outcomes. The diversity of clinical trials allows researchers to explore various aspects of NDs, leading to a better understanding of disease mechanisms and progression. Successful clinical trials can lead to the adopting of new best practices and treatment protocols, enhancing the overall standard of care for patients with NDs. Moreover, the rise in clinical trials reflects a greater collaboration among researchers, pharmaceutical companies, and funding agencies, fostering innovation and accelerating the development of new treatments.

The growing number of trials provides hope to patients and their families, encouraging greater participation in research studies and fostering a sense of optimism about future treatment possibilities. In summary, the substantial increase in clinical trials targeting NDs holds the potential to drive significant advancements in the understanding and treatment of these conditions, ultimately aiming to improve the lives of those affected.

## 3. Alzheimer’s Disease

### 3.1. Pathology and Epidemiology

**Figure 1:**
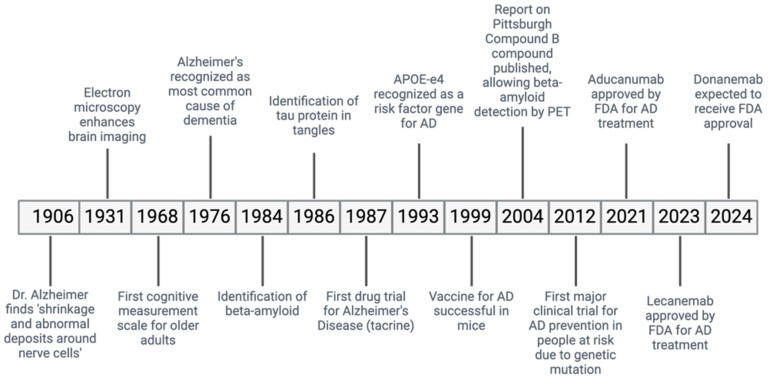
Historical timeline of AD.

Alzheimer’s disease (AD) is a ND causing a gradual decline in memory and cognitive abilities, accounting for many dementia cases worldwide (Figure 1) [13]. In AD, the accumulation of misfolded Aβ protein outside the cells and the abnormal phosphorylation of the τ protein inside the cells cause the formation of plaques and neurofibrillary tangles (NFTs), leading to the loss of neurons and synapses [14].

Diagnosing AD involves using imaging studies, such as magnetic resonance imaging (MRI), which detects the shrinkage of the hippocampus, a brain area important for memory [14]. Additionally, positron emission tomography (PET) scans are effective in detecting biomarkers of AD in the brain, such as decreased levels of the protein amyloid β42 and the presence of phosphorylated τ proteins [14].

AD is a complex and multifactorial disorder influenced by genetic and environmental factors [15,16]. It is the main cause of dementia and the fifth leading cause of mortality in the elderly population [16,17]. The global prevalence of dementia is reported to be as high as 24 million and is predicted to increase four times by the year 2050 [9]. The incidence of AD increases with age, with the prevalence of AD in people aged 65 years or older estimated to be around 10% [9]. Overall, AD is classified into early-onset or late-onset, with early-onset AD affecting 1–5% of cases and late-onset AD affecting the majority (>95%) of cases [15,16].

The exact cause of Aβ protein aggregation is unclear. Researchers suggest that genetic factors, such as mutations in the genes responsible for amyloid precursor protein (APP), presenilin 1 (PSEN 1), presenilin 2 (PSEN 2), and apolipoprotein E (APOE), increase the risk of AD [18]. Specifically, early-onset AD is often inherited and caused by mutations in the APP, PSEN 1, or PSEN 2 genes [18]. Although the function of these genes is still debatable, it is well-established that these mutations can result in an increased level of Aβ42 peptide, which can make the pathogenesis of AD more aggressive [14]. Late-onset AD is associated with mutations in the APOE gene, which also increases the risk of vascular dementia, Lewy body dementia, and other conditions [19].

Regardless of the underlying cause, the excessive production of Aβ leads to its accumulation in the form of plaques outside the cells, although the precise mechanism of its accumulation remains unclear [20]. This accumulation triggers the abnormal phosphorylation of the τ protein through dysregulated kinases such as CDK-5 and GSK-3β, which are influenced by Aβ fibril [21]. Consequently, the conformation of the τ protein changes, leading to the formation of NFTs within the neuron [22]. Aβ also activates immune responses in microglial cells through toll-like receptors, resulting in inflammation, receptor-mediated phagocytosis, and cellular clearance [23]. These mechanisms ultimately lead to a decrease in brain weight and loss of neurons, particularly in the white matter and hippocampus [14].

Presently, the FDA has approved three inhibitors of acetylcholinesterase enzymes (AChE)—donepezil, galantamine, and rivastigmine—as well as one antagonist of the (NMDA) receptor, known as memantine for AD treatment [24,25]. AChE inhibitors function by addressing the cognitive dysfunction caused by the loss of cholinergic nerves in the brain [26]. They achieve this by inhibiting AChE, which breaks down acetylcholine, resulting in increased levels of this neurotransmitter in cholinergic neurons [27]. Clinical trials have shown the effectiveness of this approach, although it may only slow down or temporarily halt cognitive decline without addressing the underlying neuronal loss and brain atrophy [28]. On the other hand, NMDA receptor antagonists prevent the excessive influx of calcium ions into neurons, which leads to excitotoxicity and cell death [24,28]. They can also counteract the neurotoxicity caused by the presence of glutamate [25]. Typically, this treatment is used for mild-to-moderate cases of AD [24]. While these treatments can provide moderate symptomatic relief, they are unable to reverse and directly affect the pathology of AD. Consequently, the need for novel AD therapies remains a priority, providing avenues for using antibodies [29].

### 3.2. Current Treatments

#### 3.2.1. Pharmacological Interventions

Pharmacological interventions remain the cornerstone of treating NDs, focusing on symptom management and disease modification. In AD, AChE inhibitors, such as donepezil, rivastigmine, and galantamine, and NMDA receptor antagonists like memantine are commonly prescribed to enhance cognitive function by modulating neurotransmitter activity [30]. However, these medications come with significant limitations. AChE inhibitors often cause side effects such as nausea, diarrhea, and insomnia, while memantine can lead to dizziness and headaches. Additionally, the costs can be prohibitive for some patients, and availability might be limited in certain regions or healthcare systems. Most importantly, their efficacy is limited to symptomatic relief, with no substantial impact on disease progression.

#### 3.2.2. Antioxidant and Anti-Inflammatory Therapies

Oxidative stress and inflammation are critical components of ND pathogenesis. Antioxidant therapies aim to reduce oxidative damage to neurons, while anti-inflammatory agents, including non-steroidal anti-inflammatory drugs (NSAIDs), target neuroinflammation to slow disease progression [31]. Despite the theoretical benefits, these therapies often lack specificity, and their side effects can be significant. For example, long-term use of NSAIDs can lead to gastrointestinal issues, cardiovascular problems, and renal damage. The costs associated with the chronic use of these medications can be substantial, and prescription regulations might limit their availability. Additionally, the efficacy of these therapies in altering disease progression remains controversial, with many studies showing limited benefits. Neurotrophic factors, which support neuron survival and function, are also being explored as potential treatments for NDs [32]. However, delivering these factors effectively to the brain poses significant challenges, and their long-term effects and safety profiles still need to be fully understood.

#### 3.2.3. Advanced Therapeutic Strategies

Advanced therapeutic strategies, such as gene therapy, stem cell therapy, and mitochondrial enhancers, are under investigation for their potential to address the underlying causes of NDs. Gene therapy aims to correct genetic defects or modulate gene expression to prevent disease progression, whilst stem cell therapy seeks to replace lost or damaged neurons [33]. Mitochondrial enhancers aim to improve cellular energy production and reduce neurodegeneration [34]. These approaches are promising but are also associated with high costs and complexities in delivery. Furthermore, their long-term safety and efficacy are yet to be determined. Availability is currently limited to clinical trial settings, and efficacy in humans remains to be fully established. Additionally, there are significant ethical and regulatory challenges associated with these advanced therapies.

#### 3.2.4. Lifestyle Interventions and Τ-Targeted Therapies

Lifestyle interventions, including diet, exercise, and cognitive training, have shown promise in reducing ND risk and progression [35]. However, the efficacy of these interventions can vary among individuals, and adherence to lifestyle changes can be challenging. Additionally, costs associated with specialized diets and exercise programs can be a barrier, and the availability of resources for cognitive training may be limited in certain areas.

Τ-targeted therapies, particularly relevant for AD, aim to prevent τ protein aggregation and the formation of neurofibrillary tangles [36]. These therapies are still largely experimental, with limited efficacy data from clinical trials. Side effects are not yet fully understood, and the costs of developing and delivering these therapies are substantial. Availability is currently restricted to clinical research settings.

#### 3.2.5. Immunotherapy and Amyloid-Directed Antibodies

Immunotherapy, specifically monoclonal antibodies, has emerged as a promising strategy for targeting pathological proteins in NDs (Table S1, Supplementary Materials). In AD, amyloid-directed antibodies such as Aducanumab, which targets aggregated forms of Aβ, have shown potential in reducing amyloid plaque burden and slowing cognitive decline [37]. The research and development of these antibodies have accelerated in recent years, with numerous trials underway to evaluate their efficacy and safety [30]. Despite mixed results in clinical trials, the continued investment in this area underscores the potential of these therapies to fundamentally alter the course of neurodegenerative diseases.

### 3.3. Monoclonal Antibodies for AD

#### 3.3.1. Bapineuzumab

Bapineuzumab is one of the earliest monoclonal antibodies developed for AD that specifically targets the N-terminal epitopes of Aβ peptides. Bapineuzumab reduces amyloid plaques in the brain by binding them and facilitating their clearance through immune-mediated mechanisms [38]. Early studies indicated that bapineuzumab reduces amyloid plaques in the brain. However, subsequent large-scale phase III trials, including trials 301 and 302, failed to show significant cognitive benefits in mild-to-moderate AD patients, highlighting important side effects [39]. The lack of clear cognitive improvement led to the discontinuation of bapineuzumab’s development. However, earlier research offered valuable insights into targeting Aβ in AD and guided the development of subsequent monoclonal antibody therapies [40].

#### 3.3.2. Solanezumab

Solanezumab is a monoclonal antibody designed to target soluble monomeric forms of Aβ peptides in AD patients, aiming to prevent their aggregation into toxic oligomers and fibrils that form amyloid plaques in the brain [41]. Initial phase II trials showed that solanezumab could reduce free Aβ in cerebrospinal fluid, raising hopes of slowing cognitive decline. However, subsequent phase III trials, specifically the EXPEDITION and EXPEDITION2 studies, failed to show significant cognitive improvement in mild-to-moderate AD patients, despite some evidence of slowing decline in mild AD cases [42]. Despite these mixed outcomes, the safety profile of solanezumab is generally favorable, with fewer incidences of amyloid-related imaging abnormalities (ARIAs) compared to other amyloid-targeting monoclonal antibodies. Despite not achieving its primary cognitive endpoints, the data from solanezumab trials contribute to a deeper understanding of the amyloid hypothesis and the challenges of translating amyloid reduction into clinical benefits [40]. Researchers continue to explore its potential in the very early stages of AD, where intervention might have a greater impact on disease progression [43].

#### 3.3.3. Gantenerumab

Gantenerumab is a human monoclonal antibody that targets Aβ plaques in AD patients. It binds specifically to conformational epitopes on the fibrillar form of Aβ, facilitating the removal of these plaques through immune-mediated processes [44]. Early studies showed that gantenerumab significantly reduces amyloid plaque burden in the brain. Clinical trials, including the SCarlet RoAD and Marguerite RoAD trials, demonstrated a notable reduction in amyloid plaques, but they yielded mixed results regarding cognitive benefits [44]. Parallel studies also failed to show a significant slowing of cognitive decline, raising questions about the direct correlation between amyloid reduction and cognitive improvement [44]. Overall, gantenerumab is well-tolerated, with few patients experiencing ARIA [44]. Ongoing research aims to optimize dosing and patient selection to enhance gantenerumab’s therapeutic potential [44].

#### 3.3.4. Crenezumab

Crenezumab is a monoclonal antibody that targets multiple forms of Aβ peptides, including monomers, oligomers, and fibrils, in treating AD. By binding to these different forms, crenezumab aims to reduce Aβ toxicity while minimizing the common side effects of amyloid-targeting therapies [45]. The antibody facilitates the clearance of Aβ through mechanisms such as phagocytosis by microglial cells, thereby potentially reducing the formation of amyloid plaques in the brain [45]. Crenezumab showed initial promise in preclinical studies and early-phase clinical trials, demonstrating its ability to lower amyloid levels and exert neuroprotective effects. However, the phase III clinical trials, including the CREAD and CREAD2 studies, did not show significant cognitive improvement in patients with mild-to-moderate AD, despite some reductions in amyloid plaque [44]. Overall, the safety profile of crenezumab is favorable, with fewer occurrences of ARIA compared to other monoclonal antibodies. The lack of significant cognitive improvement in these trials led to the cessation of further development for crenezumab [45].

#### 3.3.5. Aducanumab

Aducanumab is a monoclonal antibody that targets aggregated forms of Aβ peptides in the brain. Aducanumab binds specifically to aggregated Aβ, including soluble oligomers and insoluble fibrils, facilitating their clearance through immune-mediated mechanisms such as phagocytosis by microglial cells [46]. The goal of this treatment is to reduce amyloid plaque burden and mitigate its neurotoxic effects, potentially slowing the progression of cognitive decline in AD patients. Clinical trials of aducanumab, including ENGAGE and EMERGE studies, demonstrated that the monoclonal antibody significantly reduce amyloid plaques in a dose-dependent manner [47]. The overall data supported the potential of aducanumab to benefit patients, leading to its approval by the FDA in 2021 [47]. This approval marks aducanumab as the first AD treatment to target the underlying pathology of the disease rather than just alleviating symptoms. However, the approval process and the interpretation of clinical trial data have sparked considerable debate within the medical community, with discussions focusing on the extent of cognitive benefits and the risk of ARIA. Current studies are further evaluating aducanumab’s clinical efficacy to optimize its therapeutic use [47].

### 3.4. Ongoing Trials for Potential Therapeutics

#### 3.4.1. Lecanemab

Lecanemab is a monoclonal antibody developed to target soluble protofibrils of Aβ peptides. By specifically binding to these protofibrils, lecanemab aims to prevent their aggregation into insoluble fibrils and plaques, thereby reducing the neurotoxic effects associated with amyloid buildup [48]. Lecanemab has shown promising results in preclinical studies and early-phase clinical trials, demonstrating its ability to significantly reduce amyloid plaque burden in the brain [48]. For instance, the phase II clinical trial showed that lecanemab decreases amyloid levels and slows cognitive decline in patients with early AD, suggesting a reduction in amyloid pathology and a potential clinical benefit [49]. The safety profile of lecanemab is generally favorable, with a lower incidence of ARIAs compared to other amyloid-targeting monoclonal antibodies [48]. Ongoing phase III trials, such as the Clarity AD study, aim to further validate these findings by assessing the long-term effects of lecanemab on cognitive function and disease progression in a larger cohort of patients [50]. The development of lecanemab underscores the continuous evolution of therapeutic strategies targeting Aβ, highlighting the potential for more precise interventions that address specific pathological forms of amyloid. If successful, lecanemab could represent a significant advancement in treating AD, providing both symptomatic relief and a potential disease-modifying effect [50].

#### 3.4.2. PNT001

PNT001 is a novel monoclonal antibody that targets τ protein, which forms neurofibrillary tangles in the brains of AD patients. τ tangles disrupt neuronal function and are closely associated with disease progression and cognitive decline. Early results in targeting τ are promising in mice, but its therapeutic efficacy in humans has not yet been studied [51].

## 4. Parkinson’s Disease

### 4.1. Pathology and Epidemiology

Parkinson’s disease (PD) is a common neurodegenerative disorder, second only to Alzheimer’s disease (AD) in prevalence within the United States. Epidemiological data estimate the prevalence of PD at approximately 572 per 100,000 individuals [52]. Despite ongoing efforts, patient registries are still developing, and healthcare systems are adjusting to the rising incidence of the disease [34]. The aging population in Western countries is a significant risk factor, compounded by environmental influences and genetic polymorphisms [53]. This increasing prevalence imposes a substantial economic burden, with the total cost of neurodegenerative diseases estimated at USD 52 billion annually [52]. Globally, the number of PD cases is projected to double by 2040, with current therapies primarily addressing symptoms rather than altering disease pathology [53].

**Figure 2:**
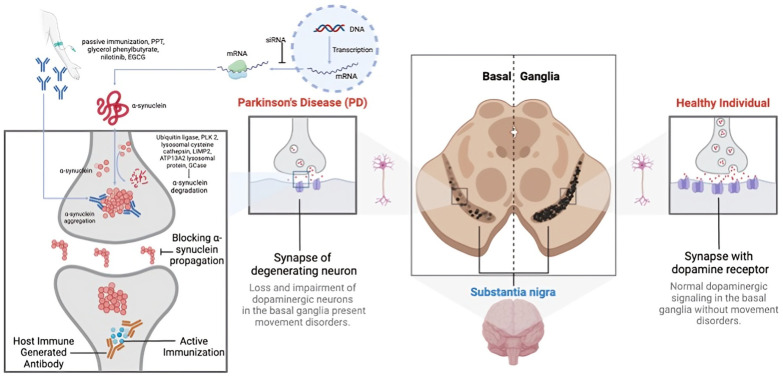
A representation of the multifaceted pathophysiology of Parkinson’s disease (PD).

PD progression is marked by the aggregation of α-synuclein (α-syn) in the brain, particularly in the substantia nigra pars compacta of the basal ganglia [54]. These aggregates, known as Lewy bodies or neurites, impair dopaminergic neurons through gain-of-function mechanisms [55]. Additionally, α-syn aggregates trigger an immune response, causing neuroinflammation and neuronal death [56]. Other proteins may also aggregate alongside α-syn, though the mechanisms remain debated [2]. The death of dopaminergic neurons exacerbates inflammation, leading to a progressive decline in motor and non-motor functions. The pathophysiology of PD involves multiple pathways, including proteasomal dysfunction and genetic factors [54]. Given α-syn’s abundance and its role in various synucleinopathies, the precise reasons for its aggregation and pathological role are still not fully understood [54,55] (Figure 2).

### 4.2. Current Therapeutics

The standard treatment for Parkinson’s disease (PD) is levodopa, a prodrug converted into dopamine upon crossing the blood–brain barrier (BBB). This conversion alleviates the symptomatic effects caused by dopaminergic neuronal loss [57]. Typically, levodopa is administered with peripheral decarboxylase inhibitors to prevent its breakdown outside the brain [57]. Despite its effectiveness, the levodopa treatment has notable side effects. Short-term use often leads to dyskinesia, and as the disease progresses, increasing the dosage of levodopa can limit its benefits and affect the patient’s quality of life. Furthermore, prolonged use may induce peripheral resistance due to the upregulation of decarboxylase enzymes, potentially limiting long-term efficacy [58].

To address levodopa-induced dyskinesia and the “OFF” time effects between treatments, adjunctive therapies like amantadine are used. Amantadine is a weak, uncompetitive NMDA antagonist that reduces dopamine reuptake, extending the effects of levodopa [59]. However, the exact mechanism by which amantadine mitigates dyskinesia remains unclear, and determining the optimal dosage is an ongoing area of research [59,60].

In addition to levodopa and amantadine, other pharmacological agents such as Monoamine Oxidase-B (MAO-B) inhibitors and Catechol-O-Methyltransferase (COMT) inhibitors are used to prolong dopamine action in the brain. These agents inhibit enzymes responsible for peripheral dopamine breakdown, thus increasing cerebral dopamine levels and alleviating PD symptoms [61]. However, they are associated with side effects, such as headaches, insomnia, and liver damage, and offer limited neuroprotective benefits against dopaminergic cell loss [62].

Surgical interventions, such as deep brain stimulation (DBS), have been approved to manage PD-induced tremors. DBS targets specific brain regions, such as the ventral intermediate nucleus (VIM) and the globus pallidus interna (GPi). Given the evolving nature of this technology, ongoing research is crucial to refine treatment protocols, identify optimal stimulation sites, and establish clear inclusion criteria [63].

Emerging therapies include active immunization strategies targeting α-syn aggregates. Preclinical studies have shown that immunizing with α-syn peptides can elicit an immune response, reducing pathological α-syn accumulation [64]. However, this approach carries the risk of autoimmune reactions, necessitating careful future research.

Induced pluripotent stem cells (iPSCs) from PD patients are used to model PD pathology and test immunotherapies. These iPSCs can differentiate into midbrain dopaminergic neurons or 3D midbrain organoids, replicating key PD features for screening antibody candidates [65]. This technology allows for genetic editing to study disease mechanisms and potential treatments.

Nonhuman primate models are also utilized in preclinical trials to better predict immunotherapy efficacy and safety. These models naturally develop age-related Lewy body pathology, providing valuable insights into the in vivo effects of antibody therapies [66].

### 4.3. Monoclonal Antibodies for PD

Research is focused on optimizing antibody design, delivery methods, and clinical trial frameworks to enhance immunotherapy’s potential (Table S2, Supplemental Materials). Strategies include engineering antibodies with improved affinity and specificity for pathological α-syn and exploring alternative administration routes to enhance brain penetration. Developing better biomarkers and clinical endpoints is crucial for accurately assessing immunotherapies’ impact on disease progression in clinical trials. Current efforts aim to refine these therapies and investigate their potential synergy with existing symptomatic treatments. Enhanced preclinical models and biomarkers are essential for transitioning these therapies from laboratory research to clinical application, offering new treatment avenues for PD.

#### 4.3.1. Cinpanemab

Cinpanemab is a monoclonal antibody that specifically targets aggregated extracellular α-synuclein. Initial phase I trials conducted in the USA involved PD patients and did not focus on measuring physiological or clinical changes but reported mild-to-moderate treatment-related adverse events (TRAEs), indicating the need for further research to assess its efficacy [67]. Subsequent phase II trials across nine countries, focusing on early-stage PD patients, did not observe significant changes in imaging biomarkers or clinical improvements. The side effects were consistent with phase I findings, highlighting the necessity for exploring alternative therapeutic approaches [67].

#### 4.3.2. Prasinezumab

Prasinezumab targets the C-terminus of α-synuclein to inhibit its transfer between neurons, a key mechanism in PD progression. Phase I trials in the USA with mild-to-moderate PD patients showed no significant cerebrospinal fluid biomarker changes, but the treatment was well-tolerated and reduced free serum α-synuclein levels, warranting further research [68]. Phase II trials across five countries with early-stage PD patients did not reveal significant imaging or clinical changes but again resulted in mild-to-moderate TRAEs, suggesting the need for larger population studies and targeted engagement tests [69].

#### 4.3.3. UCB7853

The monoclonal antibody UCB7853 targets α-synuclein and has been tested in phase I trials in the UK and the Netherlands. These trials included both healthy participants and PD patients, focusing on evaluating UCB7853’s safety and tolerability. Although no physiological or clinical changes were measured, the trials reported no adverse events, indicating a favorable safety profile. Future research should target more specific therapeutic benefits [12].

#### 4.3.4. LU AF82422

LU AF82422 targets the C-terminal of α-synuclein, enhancing regulatory T-cell activity and reducing free plasma and cerebrospinal fluid (CSF) α-synuclein levels. Phase I trials in Japan reported a decrease in α-synuclein levels, with adverse events primarily associated with lumbar punctures [70]. These promising results suggest further development is warranted.

#### 4.3.5. PRX002

PRX002 targets aggregated forms of α-synuclein to reduce free serum α-synuclein levels. Phase I trials in the USA involved healthy participants and did not assess clinical changes, but the treatment was well-tolerated, supporting continued development for PD patients [71].

#### 4.3.6. TAK-341/MEDI1341

TAK-341/MEDI1341 is being evaluated in Phase I and II trials across multiple continents. Phase I involved single IV infusions in healthy volunteers, PD patients, and multiple system atrophy patients, with various safety assessments. Phase II involves IV infusions every four weeks for one year, focusing on changes in the Unified Multiple System Atrophy Rating Scale. The ongoing trial aims to provide comprehensive data on the safety, tolerability, and efficacy of TAK-341/MEDI1341, with results expected to inform its therapeutic potential for PD and related disorders [68].

## 5. Discussion

Immunotherapy for neurodegenerative diseases, particularly AD and PD, provides a significant shift towards targeted and potentially disease-modifying treatments. The clinical trials reviewed in this paper highlight both the promise and challenges associated with these approaches.

In both Alzheimer’s disease (AD) and Parkinson’s disease (PD), antibody-based therapies targeting pathological aggregates, such as amyloid-β (Aβ) and α-synuclein (α-syn), have shown potential in reducing biomarkers like amyloid plaques and pathological aggregates. However, translating these biomarker changes into consistent clinical benefits remains challenging. For AD, antibodies like aducanumab and lecanemab have reduced amyloid plaques, but cognitive improvements have been inconsistent and modest, as evidenced by the controversial FDA approval of aducanumab [72]. Similarly, in PD, antibodies like prasinezumab have demonstrated biomarker reduction in early trials, yet clinical benefits are elusive [69]. This highlights the critical need for robust, long-term studies to definitively link biomarker reductions to meaningful clinical outcomes and a deeper understanding of the mechanisms driving these improvements. Both fields underscore the necessity of reliable biomarkers that accurately reflect disease progression and treatment response, and comprehensive research to bridge the gap between biomarker changes and tangible clinical benefits [73].

The limitations for using monoclonal antibodies in immunotherapy are significant and include several challenges that must be carefully managed. These limitations encompass potential adverse effects such as ARIAs and the variability in patient responses [74]. The safety profile of these treatments requires careful consideration and ongoing monitoring for adverse effects is crucial to ensure patient safety and optimize treatment outcomes [48].

Moreover, the heterogeneity of AD and PD suggests that therapies targeting a single protein may be insufficient. In AD, tau protein aggregation, along with amyloid aggregation, is increasingly recognized as a crucial contributor to neurodegeneration. In PD, genetic mutations and mitochondrial dysfunction extend beyond alpha-syn aggregation and play vital roles in disease progression. This complexity necessitates the development of combination therapies that can address multiple pathological mechanisms simultaneously. For example, combining Aβ-targeting monoclonal antibodies with those targeting τ could provide a more comprehensive approach to treating AD [73]. In PD, therapies that address a-syn aggregation and mitochondrial dysfunction may prove more effective [67].

Future research should focus on improving therapies to enhance specificity and reduce side effects. Researchers may consider developing antibodies with higher affinity and better brain penetration, creating bispecific antibodies that target multiple pathological proteins and reducing immunogenicity to minimize adverse effects [48]. Studies should also aim to develop reliable biomarkers for early diagnosis and treatment monitoring, including neuroimaging, cerebrospinal fluid, and blood-based markers [7]. Enhanced imaging techniques to detect early brain changes can enable earlier intervention. Additionally, using precision medicine to tailor treatments based on individual genetic profiles and disease characteristics will be crucial [73]. Personalized treatment plans considering a patient’s specific genetic mutations, disease stage, and comorbid conditions could improve outcomes and reduce adverse effects [30]. Leveraging advancements in genomics and bioinformatics can help identify patient subgroups that are more likely to benefit from specific therapies. Exploring combination therapies, such as combining immunotherapy with small molecules, gene therapy, and stem cell approaches, can address both symptoms and underlying causes, offering a holistic treatment strategy.

Continued investment in large-scale, long-term clinical trials is essential to gather robust data on the safety and efficacy of monoclonal antibodies. These trials should incorporate diverse patient populations and real-world settings to ensure the broader applicability of the findings [47]. Moreover, real-world evidence and post-marketing surveillance will be crucial for understanding the long-term effects of these therapies and identifying any rare or delayed adverse effects [7].

Finally, the socio-economic aspects of these therapies must be addressed. The high cost of monoclonal antibody treatments presents a significant barrier to access, particularly in low- and middle-income countries [9]. Efforts should be made to develop cost-effective production methods and ensure these therapies are affordable and accessible to all patients. Policymakers, healthcare providers, and researchers must collaborate to address these barriers and ensure that advancements in treatment reach the populations that need them most [11].

While current immunotherapy for AD and PD face significant challenges, ongoing research and emerging strategies holds the potential to transform the treatment landscape. By addressing these limitations through innovative approaches and continued investment in research, there is hope for more effective and personalized interventions in the future.

## 6. Conclusions

Neurodegenerative diseases like AD and PD are growing public health crises with significant socio-economic implications. Driven by an aging population, the rising incidence of these diseases demands urgent advancements in therapeutic strategies. Despite increased clinical trials and research on immunotherapy and the use of monoclonal antibodies, a definitive cure remains elusive. Future efforts should focus on improving therapies, developing biomarkers for early diagnosis and treatment monitoring, and addressing socio-economic barriers to care. By integrating these approaches, we postulate a reduction in the prevalence and impact of AD and PD, improving the quality of life for millions affected.

## Data Availability

No new data was created in this study. The data that support the findings of this study are included within the article and Supporting Materials.

## Supplementary Materials

The following supporting information can be downloaded at: https://www.mdpi.com/article/10.3390/cells13181527/s1, Table S1: Monoclonal antibodies trialed for AD [42,45,51,73,75,76], Table S2: Monoclonal antibodies trialed for PD [69,70,71,77,78,79].
