# Supplementary material for: Immunotherapy for Parkinson’s Disease and Alzheimer’s Disease: A Promising Disease-Modifying Therapy
Source: Cells. 2024 Sep 12;13(18):1527. doi: 10.3390/cells13181527 (PMC11429902; doi:10.3390/cells13181527)
Supplement: Supplementary file 1 [file cells-13-01527-s001.zip › cells-3143741-supplementary.pdf]

Table S1. Monoclonal antibodies trialed for AD.

Monoclonal Antibody Name	Phase	Country	Study Type	Participants	Target	Dosage	Duration (weeks)	Mechanism of Action	Physiological Changes	Clinical Changes	Side Effects	Future Directives	Reference
Bapineuzumab	III	USA, Canada, Germany, Austria	Randomized 3:2, double-blind	Age 50-88 years, met AD criteria, MMSE 16-26, APOE ε4 carriers	N-terminal region of Aβ	0.5 mg/kg	78	Reduce Aβ accumulation	Reduced amyloid accumulation in APOE4 carriers	No clinical benefit	Similar AE incidence	Repeat phase III trials, higher doses	[75]
Bapineuzumab	III	USA, Canada, Germany, Austria	Randomized 3:3:4, double-blind	Age 50-88 years, met AD criteria, MMSE 16-26, APOE ε4 carriers	N-terminal region of Aβ	0.5-1 mg/kg	78	Reduce Aβ accumulation	Reduced amyloid accumulation in APOE4 carriers	No clinical benefit	Similar AE incidence	Repeat phase III trials, higher doses	[75]
Crenezumab	III	30 countries	Randomized, double-blind	Age 50-90 years, met AD criteria, MMSE 18-28	Mid-region of Aβ (residues 11-25)	60 mg/kg every 4 weeks	100	Target Aβ oligomers	No meaningful biomarker changes	No clinical benefit	No significant difference in AEs	Longer duration trial	[45]
Crenezumab	III	27 countries	Randomized, double-blind	Age 50-90 years, met AD criteria, MMSE 18-28	Mid-region of Aβ (residues 11-25)	60 mg/kg every 4 weeks	100	Target Aβ oligomers	No meaningful biomarker changes	No clinical benefit	No significant difference in AEs	Longer duration trial	[45]
Gantenerumab	III	15 countries	Randomized, double-blind	Age 50-90 years, met AD criteria, MMSE 16-26	Aβ plaques	Increasing dose 120-510 mg q4w & q2w	36	Amyloid-plaque removal via phagocytosis	Dose-dependent amyloid reduction	Reduced amyloid PET, clinical decline	Higher incidence of AEs	Investigate AE causality	[73]
Solanezumab	III	Australia, Canada, Japan, USA	Randomized, double-blind	Age 65-85 years, no dementia, elevated brain amyloid	Monomeric Aβ	1600 mg IV q4w	240	Increase Aβ clearance from brain	Amyloid accumulation continued	No cognitive benefit	Similar AE incidence	Greater racial diversity, COVID impact	[42]
Lecanemab	II	11 countries, 3 continents	Randomized, double-blind	Amyloid-positive, episodic memory impairment, MMSE ≥22	Soluble Aβ protofibrils	2.5-10 mg/kg q2w or q4w,	78	Bind Aβ protofibrils, promote clearance	Reduced brain amyloid, CSF biomarker changes	20% reduced clinical decline	Well tolerated	Further phase III studies	[73]

Aducanumab	III	Global	Randomized, double-blind	Age 50-85 years, mild cognitive impairment or mild dementia due to AD	Aggregated Aβ forms	3-10 mg/kg	78	Target soluble/insoluble Aβ oligomers/fibrils	NA	NA	Well tolerated	Longer trials, diverse population	[76]
PNT001	I	USA	Randomized, double-blind	Age 21-65 years, no medical risks	cis-pT231 τ	1000-4000 mg	12	Bind cis-pT231 τ, interrupt τ effects	CSF antibody exceeded τ binding levels	NA	Well tolerated	Next phase, more patients	[51]

Table S2. Monoclonal antibodies trialed for PD.

Monoclonal Antibody Name	Phase	Country	Study Type	Participants	Target	Dosage	Duration (weeks)	Mechanism of Action	Physiological Changes	Clinical Changes	Side Effects	Future Directives	Reference
Cinpanemab	II	9 countries	Randomized, double-blind	Early-stage PD	Aggregated extracellular α-synuclein	250-3500 mg every 4 weeks	52	NA	No imaging biomarker changes	No clinical changes	Mild/moderate TRAE	Utility appears limited, other approaches needed	[77]
Prasinezumab	II	5 countries	Randomized, double-blind	Early-stage PD	C-terminus of α-syn	1500-4500 mg every 4 weeks	52	Inhibit neuron-to-neuron α-syn transfer	No imaging changes	No clinical changes	Mild/moderate TRAE	Larger population, target engagement tests needed	[69]
UCB7853	I	UK, NL	Randomized, double-blind	Healthy, PD	α-synuclein	Single/multiple IV infusions	188	NA	NA	NA	NA	NA	[78]
LU AF82422	I	Japan	Randomized, double-blind	Healthy, PD	C-terminal of α-syn	75-9000 mg single IV infusion	157	Enhances Treg function	Reduced free plasma/CSF α-syn	Lowered α-syn	Mainly from LP	Appropriate for further development	[70]

PRX002	I	USA	Randomized, double-blind	Healthy	α-synuclein	0.3-30 mg/kg single IV infusion	16	Targets aggregated α-syn	Reduced free serum α-syn	Not assessed	Well tolerated	Supports continued development in PD patients	[71]
TAK-341/ MEDI1341	I, II	USA, North America, Europe, Asia	Randomized, double-blind	Healthy volunteers, Parkinson's patients, Multiple system atrophy patients	C-terminal epitope on monomeric and aggregated α-synuclein	Phase I: Single IV infusion (healthy), 3 doses in 8 weeks (PD). Phase II: IV infusion every 4 weeks for 1 year	Phase I: 3 months (healthy), 21 weeks (PD). Phase II: 1 year	NA	NA	NA	Phase I: Added vision/eye assessments (healthy), adverse events, safety, ophthalmic, cognitive/psychiatric assessments (PD)	Phase I (healthy) completed March 2021; Phase I PD ended after 2 dose cohorts. Phase II ongoing through Aug 2025, primary outcome change in Unified Multiple System Atrophy Rating Scale.	[79]
